# Improving Working Conditions and Job Satisfaction in Healthcare: A Study Concept Design on a Participatory Organizational Level Intervention in Psychosocial Risks Management

**DOI:** 10.3390/ijerph17103677

**Published:** 2020-05-23

**Authors:** Cristina Di Tecco, Karina Nielsen, Monica Ghelli, Matteo Ronchetti, Ivan Marzocchi, Benedetta Persechino, Sergio Iavicoli

**Affiliations:** 1 Department of Occupational and Environmental Medicine, Epidemiology and Hygiene, Italian National Workers Compensation Authority, via Fontana Candida, Monte Porzio Catone, 00078 Rome, Italy; c.ditecco@inail.it (C.D.T.); m.ghelli@inail.it (M.G.); i.marzocchi-sg@inail.it (I.M.); b.persechino@inail.it (B.P.); s.iavicoli@inail.it (S.I.); 2 Institute of Work Psychology, Sheffield University Management School, University of Sheffield, Sheffield S10 2TN, UK; k.m.nielsen@sheffield.ac.uk; 3 Department of Psychology, Sapienza University of Rome, Via dei Marsi, 78, 00185 Rome, Italy

**Keywords:** occupational safety and health, working conditions, psychosocial risks, job satisfaction, work-related stress

## Abstract

This paper contributes to the literature on organizational interventions on occupational health by presenting a concept study design to test the efficacy of a Participatory Organizational-level Intervention to improve working conditions and job satisfaction in Healthcare. The Participatory Organizational-level Intervention is developed using the Italian methodology to assess and manage psychosocial risks tailored to Healthcare. We added an additional step: evaluation, aiming to examine how the intervention works, what worked for whom and in which circumstances. This ongoing study is conducted in collaboration with two large Italian hospitals (more than 7000 employees). The study design comprises a quasi-experimental approach consisting of five phases and surveys distributed pre- and post-intervention aiming to capture improvements in working conditions and job satisfaction. Moreover, to evaluate the efficacy of the Intervention in terms of process and content, we use a realist evaluation to test Context-Mechanisms-Outcome (CMO) configurations. We collect contextual factors at baseline and during and post-intervention process data on the key principles of line manager support and employees participation. This study is expected to provide insights on methods and strategies to improve working conditions and employees’ job satisfaction and on national policies in the occupational health framework.

## 1. Introduction

Working conditions are changing rapidly. The increased labor market flexibility, the advancement in technology, and changes in workforce characteristics are changing the way work is designed and organized [1]. Work-related psychosocial risks are defined as those risks in the workplaces linked to the design and management of work, as well as its social and organizational contexts that are considered the key emerging risks to well-being since they are linked to workplace problems, such as work-related stress [2]. Managing psychosocial risks in organizations is linked to the improvement in working conditions since it aims to identify and intervene on aspects of work and work organization that may constitute a risk for employees’ stress levels and well-being. This management can be done on the basis of a risk management paradigm, starting with the identification of problems and an assessment of the risks associated and then identifying best solutions to reduce such risks at the source [2].

Legal requirements to manage psychosocial risks have been a strong determinant for organizations taking measures to psychosocial risk management [3]. In the European Union, the Framework Directive 89/391/EEC on Safety and Health of Workers at Work established employers’ general obligations to ensure employees’ health and safety by addressing all types of risk in a preventive manner, including the management of psychosocial risks [4]. To further support psychosocial risk management at the European level, a Framework Agreement on work-related stress has been introduced [5]. The EU Member States have implemented these frameworks in different ways and to varying degrees. In Italy, the requirements are higher than EU law, and there is a direct reference to work-related stress [6,7]; however, at present, the focus of the national strategy has been on assessing risks rather than developing a national strategy that is more practically oriented to managing psychosocial risks, reduce stress, and improve well-being.

In the present paper, we describe the design of a pilot study to managing psychosocial risks in the healthcare sector through a Participatory Organizational-level Intervention (POI). Taking an approach from risk assessment to risk management, the POI aims to improve working conditions and employees’ job satisfaction.

According to previous research, improvements in job satisfaction have been linked to significant changes in working conditions during participatory interventions [8,9]. Participation has shown an effect on employees’ motivation since it increases job control, the awareness about work organizations and roles into the organization, the involvement in workplace problems, and the understanding about work processes. Thus, in participatory process interventions, workers’ motivation and engagement is linked to improvements in psychosocial working conditions, and it is in turn linked to improvements in job satisfaction [8,9]. Job satisfaction has a strong association with workers’ health. In a meta-analysis of almost 500 studies with more than 250,000 employees from a wide range of organizations, self-reported job satisfaction showed a strong link with employees’ wellbeing and health, particularly with mental health/psychosocial problems [10]. Thus, interventions that have an impact on job satisfaction may produce benefit to employees’ health too, with particular regard to mental health and wellbeing. Accordingly, we focused on improvements on job satisfaction as a general measure of employees’ well-being at work and as the most used indicator in participatory interventions on working conditions and work organization [11]. If successful, this approach will be integrated into the existing strategies and support the improvement of the Italian policy framework.

Psychosocial risk management is crucial to ensure worker health and wellbeing [2,12]. In Europe, 25% of employees have experienced work-related stress [13]. Psychosocial risks have been linked to cardiovascular diseases, musculoskeletal disorders, poor job satisfaction, high turnover, and mental ill-health [13,14,15,16].

Despite the EU framework, findings from the third wave of the European Survey of Enterprises on New and Emerging Risks [17] showed that 21% of the EU28 perceived psychosocial risks to be more challenging to manage than other Occupational Health and Safety (OSH) risks. Acknowledging this challenge, it is crucial that organizations are supported in managing psychosocial risks, through approaches and practical tools. Such support is of particular importance in high risk sectors. The healthcare sector has received particular attention in relation to psychosocial risks in the past two decades, since stress levels among healthcare personnel are higher compared to other sectors [18]. Working in very demanding environments may have detrimental effects on the quality of care for patients and relatives [19,20,21]. The sixth European Working Conditions Survey [18] highlighted that employees in the healthcare sector are exposed to the highest levels of work intensity, resulting from excessive quantitative and emotional demands, and are often subject to adverse social behaviors. Moreover, compared to other sectors, a higher percentage of healthcare employees reported insufficient time to carry out their job, frequent disruptive interruptions, emotional hiding, handling with angry patients or their relatives, and living emotionally disturbing situations three-quarters or more of time [18]. A similar trend came from INSuLa, the national Italian survey of employees’ perceptions of health and safety [22]: across nine sectors studied, employees in the healthcare sector reported the highest levels of work-related stress.

According to the second European survey of enterprises on new and emerging risks [3], the sector “health and social work” extends great efforts in managing psychosocial risks, both in favorable and unfavorable national contexts; important drivers of these initiatives are the desire to increase commitment and employees’ and managers’ involvement. Further improvements are nevertheless required, particularly in evaluating the effectiveness of risk assessment and management interventions. Findings from INSuLa revealed that, according to employers, organizational-level interventions in the healthcare sector were ineffective in 10.6% of cases [22]. One challenge to developing effective organizational-level interventions is that currently many interventions are evaluated in terms of whether the interventions worked or not, if evaluated at all. In order to ensure that learning from one intervention is transferred to other settings, we need to evaluate interventions asking the questions of what works for whom in which circumstances [23]. Such rigorous evaluation is currently lacking [24]. Answering these questions is crucial for policy makers to develop national policies for psychosocial risk management across occupational contexts.

In the present paper, we present a concept study design outlining the Italian Workers Compensation Authority (INAIL) approach to managing psychosocial risks. In its first stages, the focus of the INAIL methodology was to assess psychosocial risks. This stage has been scientifically validated [25,26,27]. We are now entering the second stage, where methods and tools to support organizations taking corrective actions to eliminate or minimize risks, and evaluating their effectiveness, need to be developed and validated. The approach will be piloted in the healthcare sector. In the following, we describe in detail the second stage approach to managing psychosocial risks.

Our approach employs participatory strategies to engage different key stakeholders (employer, line managers, and occupational safety and health (OSH) professionals), as well as employees as active agents in the change process [11,28]. Using this participatory approach, OSH professionals, employees and their managers collectively identify psychosocial risks, as well as identify and develop tailored solutions to improve their working conditions.

The purpose of this paper is to describe the background, study design, intervention approach, and evaluation methods for an Italian case study on the management of psychosocial risks in the healthcare sector. By using a rigorous research design to test an evidence-based model, including validated tools in large Italian healthcare organizations, this study will contribute to knowledge on interventions to prevent work-related stress in two ways. First, we aim to provide a structured process, with proven effectiveness and tailored to the specific needs of the healthcare sector, and to contribute practically to national policies on the management of psychosocial risks at work.

Second, we aim to investigate the role that some key principles, i.e., intervention fit, employee participation, and management support, play in reducing psychosocial risks and improving working conditions and job satisfaction as a measure of employees’ well-being in a POI.

### 1.1. The INAIL’s Methodology

Italian OSH’s regulatory framework (Leg. Decree 81/2008 and subsequent additions and amendments) has specifically stated the obligations to assess and manage risks associated with work-related stress in accordance to the 2004 European Framework Agreement. More guidelines have followed in 2010 representing the minimum level of implementation published by the Ministry of Labor.

INAIL in its role of the Italian Workers’ Compensation Authority operates the national system of compulsory insurance for workers and prevention against accidents at work and occupational diseases. INAIL is also the leading National Research Institute in the field of Occupational Health and Safety and aims to timely address emerging issues and risks for workers’ health. Through the Department of Occupational and Environmental Medicine, Epidemiology and Hygiene (DiMEILA), INAIL is committed in developing knowledge and providing solutions to address traditional occupational risks, as well as risks emerging, including psychosocial risks at work.

According to its mandate, in 2011, INAIL published a first methodological proposal for the assessment and management of psychosocial risks at work to support organizations in answering to the legal obligation by the means of sustainable and evidence-based approaches [29]. This first edition mainly focused on psychosocial risk assessment. In 2017, INAIL published a second edition more concerned on the management of risks, and current research is oriented to gain a deeper understanding of the tools and methods that may ensure effective psychosocial risk management [30].

The INAIL approach constitutes a multi-phase organizational intervention employing a participatory process to mitigate the negative impact of adverse working conditions on aspects related to job satisfaction—from the screening and the assessment of risks to the identification and implementation of the best corrective and preventive actions [26]. The INAIL methodology is originally comprised of 4 phases to assess and manage risks for work-related stress to identify the best fitting and effective actions for work-related stress prevention. The first phase is Preparation, in which the POI process is planned and strategies to ensure commitment at all levels within the organization are developed. The second and the third phases are two risk assessment phases where psychosocial risks are assessed through objective and verifiable indicators of work-related stress and by surveying employees’ perceptions of psychosocial risk factors. The method offers two validated assessment tools: INAIL’s Checklist and the Management Standards Indicator Tool [31,32,33]. The fourth phase is Actions and monitoring, which aims to identify actions based on risk assessment findings and to develop (and monitor) action plans. Since this process is cyclical and requires the evaluation of the POI’s effectiveness before starting a new cycle of risk management, we include a fifth phase aimed to examine not only if the intervention works, but also what worked for whom in which circumstances.

INAIL’s methodology represents an immediate evidence-based easy-to-use solution allowing Italian organizations to meet legal requirements and can be used in different sectors. The INAIL methodology is modular by nature allowing the inclusion of sector-specific surveys/questionnaires for identifying risks. Indeed, many psychosocial risks are common to most of sectors [34,35], but some risks are specific to some sectors or activities; these specific risks should be integrated into the risk assessment. The present study focuses on the INAIL methodology tailored to the healthcare sector to improve working conditions and job satisfaction through feasible and appropriate actions.

### 1.2. Key Principles of an Participatory Organizational-Level Intervention

A recent review of POIs revealed three key principles of the intervention process and its supporting context [36]: employee participation, senior management and line management support, and fitting the intervention to the organizational context. If not considered adequately, they may impact adversely on the intervention’s outcomes [25,36].

To ensure a successful intervention outcome, tailoring or fitting the intervention to the organizational context is crucial (e.g., size of organization, productive sector, workforce specificities, goals, and mission of the organization) [36,37]. This is in line with the risk management paradigm that calls for the identification of the specific risks into the workplace and the implementation of fitting interventions [34,35]. There is also evidence to suggest that POIs have a better chance of having a positive impact when there is employee involvement and participation [11,38]. Participation can be achieved in different ways. Most POI approaches suggest the use of steering groups to ensure employee participation, directly or through their representatives for health and union representatives. Other ways are the involvement of employees in the screening of risks and/or including their input to action plans through participative tools as focus groups on risk assessment findings [38]. A previous study on the psychosocial risk management [25] using the INAIL’s methodology evidenced that skipping some key aspects of the process during the POI may result in false positive in the risk assessment results. Such findings suggested that aspects as the involvement and support of key stakeholders, such as employees, OSH professionals, and managers, was crucial for having reliable findings and defining fitting and effective actions.

When implementing changes in work practices and procedures, employees and managers play an active role since such changes require they change their behaviors [38,39]. For this reason, their active involvement and participation in understanding and acknowledging which changes need to be introduced is essential in POIs. It has been suggested that senior and line managers play an important role in translating plans in actions because they are responsible of organizational changes [40]. Accordingly, in this kind of interventions, the line managers’ support may help ensure employees’ participation and commitment, as well as guarantee employees are motivated to make the necessary changes to working conditions [39]. In the present pilot study, we adopt an evaluation approach to investigate the role of these key principles (namely participation, management support, and intervention fit) play in ensuring a POI process that leads to improvements in working conditions and job satisfaction.

## 2. Material and Methods

### 2.1. Design and Setting

**Figure 1:**
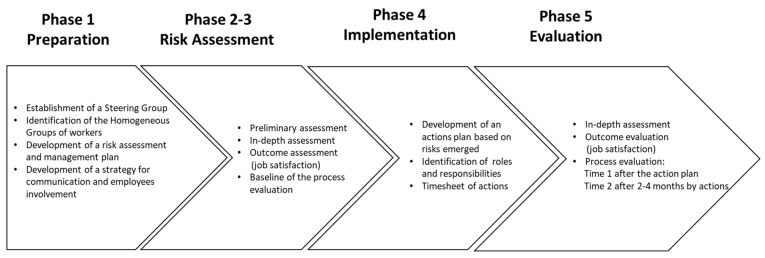
Organizational intervention on Occupational Health framework.

The study design comprises a quasi-experimental approach consisting of surveys distributed pre-, during and post-intervention [39]. At baseline and follow-up, working conditions and job satisfaction will be measured. At baseline, we also include contextual factors to capture the principle of intervention fit [36]. During the intervention and post-intervention, we will collect intervention and post-intervention process data on the key principles of line manager support and employee participation; we will also collect information about the actual changes implemented (see Figure 1).

### 2.2. Study Population, Sample, and Procedures

**Figure 2:**
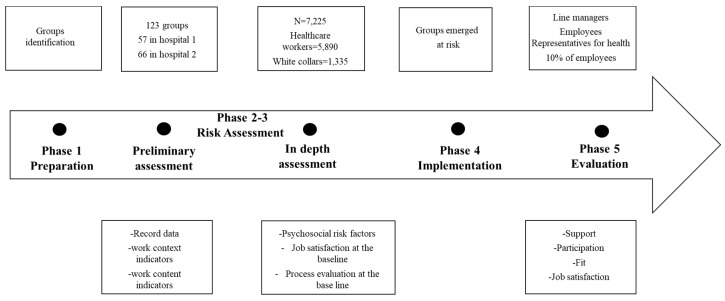
Design and sample.

This study will take place in two large Italian hospitals comprising 7225 employees, respectively, one from the Centre of Italy with 3647 employees and one from the North with 3578 employees, where 81% are healthcare providers and 19% are white-collar. The POI involves all employees in both hospitals. As explained in depth below, employees are divided in units in the POI to allow a more effective assessment and action implementation. One hundred twenty three groups have been identified (57 and 66 groups, respectively, in each hospital). All groups are involved in the five phases as shown in the Figure 2. All groups identified during the Preparation Phase are subjective to risk assessment. Record data and objective indicators related to work content and context are collected and assessed for each group. Then, employees of all groups are involved in the survey on Psychosocial risk factors, Job Satisfaction, and Process Evaluation at the baseline. Data collection involves all employees from each group and data are analyses at group and organizational levels. Based on findings of the risk assessments, an action plan is developed for groups emerged at risks. After, two rounds of the Evaluation Phase are planned, the first immediately after the action plan development and the second after 2–4 months by the actions implementation. Line managers, the 10% of employees, and their union representatives, who are also responsible for health and well-being in Italy, are surveyed on the intervention process (participation, management support, and intervention fit). Data collected in the Evaluation phase are analyzed at the group and organizational level. As outcome evaluation, employees are also surveyed on psychosocial risks and job satisfaction.

### 2.3. The Participatory Organizational-level Intervention

This study is based on the INAIL’s methodology for the assessment and management of psychosocial risks tailored to the Healthcare sector [26,41,42]. The INAIL’s methodology provides four main phases: 1. Preparation, 2. Preliminary Assessment, 3. In-depth assessment, and 4. Actions and monitoring [26]. In this study, a fifth phase was added, named Evaluation, to investigate the effectiveness of the POI by answering the questions of what works for whom in which circumstances. A full description of each phase is provided below.

*Phase 1: Preparation*

Preparation is a starting phase for the POI where organizations plan the intervention process and develop strategies for how to ensure commitment to the process at all levels within the organization. This involves some essential steps useful for process, such as identifying persons and roles involved, planning activities, and procedures. The main steps of this phase can be summarized as follows:

1. Establishment of the Steering Group for the assessment and management of psychosocial risks. The employer, or in the case of hospitals, senior management, formally establishes a Steering group consisting of key stakeholders with a stake in worker health and working conditions, such as OSH professionals, human resource managers, and occupational psychologists. The key function of the Steering Group is to plan, monitor, and facilitate the process, to determine key milestones and to inform employees about intervention progress. A project champion should be identified who can coordinate the different activities and roles [43].
2. Identification of the Homogeneous Groups of employees. Following Italian recommendation [30], organizations with more than 30 employees must set the assessment and management of work-related stress risks at the group level. As the hospitals involved in the present study have more than 3000 employed each, the Steering Committees in the respective hospitals identify Homogeneous Groups. Such groups consist of employees who share similar aspects of work organization, thus experiencing similar working conditions and work environment, who are within the same chain of command, and who receive communication through the same channels.
3. Development of a POI plan with a formal timesheet of each action to be taken, roles, resources, and responsibilities.
4. Development of a strategy of communication to inform employees about the POI, its phases and framework, surveys/questionnaires used in the assessment, figures involved, and timesheet. Communication should happen using the organization’s formal communication channels to disseminate information in the organization. Moreover, employee representatives for health are involved to improve informal communication, too [43].

*Phases 2–3: Risk Assessment*

Two assessment phases are conducted: (1) a preliminary assessment based on objective indicators related to the work content and context, as well as organizational record data as main indicators of work-related stress named sentinel events; (2) an in-depth assessment based on employees’ perceptions of psychosocial risks. For such risk assessment phases, the “standard methodology” offers two validated tools, respectively, an organizational Checklist (I-Check) [31] and a standardized questionnaire (the Italian version of the Management Standards Indicator Tool) [32,33]. At a glance, the I-Check measures trends in some organizational data named sentinel events that may be signal of work stress (such as sickness absences, turnover, injuries at work, etc.) and verifiable indicators of the work content and context. The Management Standards Indicator Tool measures seven dimensions of the psychosocial work environment.

The tailored methodology for the healthcare sector adds some organizational data (e.g., concerning patients) and scales to capture the sector-specific psychosocial risks. These are based on a preliminary proposal developed by the University of Bologna in a project led by INAIL and financed by the Ministry of Health in 2016 [41,44]. Risk assessment was developed after a literature review and based on the findings of a workshop involving main stakeholders of OSH management in this sector using a participatory approach. This included: (1) adding to the risk assessment further and tailored indicators and dimensions to catch distinctive psychosocial risks of the healthcare work; (2) developing specific indications and procedures to apply the methodology in a healthcare context; (3) providing tools to translate assessment findings in fitting actions and interventions; and (4) developing procedures to evaluate the effectiveness of the whole POI process. After a first test, the proposal received a revision, and the final version is object of this study. A full list of standard and integrative indicators and measures tailored to healthcare sector are reported in Table 1 and Table 2.

A measure of satisfaction with job [45] was included as baseline of the outcome evaluation. Data will be collected at group level and analyses at group and organizational levels.

*Phase 4: Actions Plan and Implementation*

This phase provides two main actions: the identification and planning of corrective and preventive actions and the implementation of actions identified.

The identification of best and fitting actions is based on findings of the assessment phases where psychosocial risks are identified as a way to improve working conditions and job satisfaction. Moving from assessment to action is a crucial step that requires a reflection on which changes the way work is organized, designed, and managed may improve working conditions.

The Steering group has the responsibility for the planning of corrective actions on the basis of assessment findings but can involve other key stakeholders in light of the participatory approach to ensure the use of relevant expertise to understand which changes are needed and most efficiently implemented.

Steps recommended for moving from the assessment to the identification and plan for actions are detailed below. For each step, practical tools and templates for moving from one step to the next of the process will be developed to support the Steering group in the identification, planning, and implementation of actions and to ensure a consistent approach across units.

1. Identifying corrective/improvement action priorities based on the assessment results. The Steering Group examines and discusses the results of the assessment in order to establish the priority areas in which to intervene, especially in the event of multiple critical aspects requiring different actions.
2. Verifying the need for any in-depth analysis or additional information. At this stage, participation is crucial. The involvement of the workers from the group, through focus group and workshops, can be helpful to better interpret the results of the assessment phases and to gather suggestions for effective and appropriate solutions. Focus groups can be conducted to inform the work of the Steering group in identifying corrective actions, particularly in those groups where it is not immediate to link actions to the risk areas emerged.
3. Establishing improvement actions with regard to the priorities identified. The Steering Group establishes the actions to be implemented by evaluating their relevance and feasibility. At this stage, involvement of line managers with operational meeting is suggested. Line managers have a decision-making power over work processes and procedures requiring changes. They have also a role in promoting and communicating changes to employee. They can support and facilitate the implementation of the improvement actions defined by the Steering Group.

Once actions have been identified, the Steering group agrees a plan of actions including how these may be realistically integrated into existing work practices and procedures. Action plans will detail who will be responsible for implementing actions and other key stakeholders who may support the implementation of corrective actions (human resource, line managers, specific employees, internal psychologists, etc.). In the action plan, activities and tools identified for monitoring and evaluating the effectiveness of corrective actions must be set out. The plan for actions will be shared and discussed with line managers, and employees must, in turn, be informed about which changes will be introduced, why, and how to ensure they buy into and are ready to change their work behaviors that actions require.

The implementation phase mirrors the action plans identified in terms of time, roles, and responsibilities identified and plan for monitoring and evaluating the effectiveness of corrective actions.

According to the recent development in occupational health psychology, we focus on the Individual, the Group, the Leader, and the Organizational level model (IGLO model) [46,47]. This model calls for actions to be developed at all the four levels and for the active involvement of each level in the intervention activities. Taking as its starting point the risk assessment findings, an action plan will be developed that identifies which actions need to be taken at each level to improve working conditions and job satisfaction but also what support is needed at each level to ensure sustainable change.

At the Individual level, employees should consider which actions they can take as individuals. Employees and their representatives for health (which are the union representatives, too, in Italy) are active involved in the assessment activities, actions plan, and process evaluation, through questionnaires and focus groups. At the Group level, actions may be implemented to promote a good social climate. Risk assessment and process evaluation are analyzed at group level. At the Leader or line manager level, training line managers in how to promote good working conditions at work may be necessary. As described above, line managers has also an active role in action plan and contribute to the implementation supporting the changes. Finally, at the organizational level, actions can be taken to develop supportive policies, e.g., flexible working or rotas, that promote work-life balance. Actions will also consider the existing organizational resources, procedures and facilities to hinder the implementation of the organizational intervention. Moreover, an evaluation of the process will also be conducted at the organizational level through an audit tool, including all the steps favoring the effective development of the POI.

*Phase 5: Evaluation*

To ensure that evaluation will inform future POIs and policy making, the pilot project employs a realist evaluation approach. This approach seeks to answer not only the question of whether an intervention worked, but answering the questions of what worked for whom in which circumstances [48] through the examination of Context-Mechanisms-Outcome (CMO) configurations. These configurations examine what the contextual factors are that may either facilitate or hinder that a mechanism is being triggered and what the outcome is when a mechanism is being triggered [48].

Mechanisms are the working ingredients in terms of the intervention process (e.g., participation, management support, and intervention fit) and the content of the intervention (actions taken at the IGLO levels), and outcomes are any improvements in working conditions and job satisfaction. We include three contextual factors, which may influence whether the intervention’s mechanisms will be triggered. First, the extent to which the intervention fits with the goals of the healthcare organizations. If the intervention does not align with the goals of patient care and is not seen to be appropriate for successfully addressing the psychosocial risks [49], then employees and managers are unlikely to engage in the intervention process. Second, an important precursor to change is the extent to which employees and managers understand the goals of change. Previous research has found that poor communication about an intervention’s goals and objectives prevents employees and managers engaging in the participatory process [50]. Third, participants’ readiness for change is crucial for mechanisms to be triggered. The extent to which participants are confident that their engagement in the intervention can lead to improvements in working conditions and that they are ready to accept changes introduced as a result of the intervention [51] will determine their actual making changes and engaging in the intervention process, thus enabling the mechanisms to be triggered.

At baseline, the preliminary assessment and the in-depth assessment will be conducted (see Figure 1) together with measures of context on all employees from each group. Around 6 months into the project, process measures of participation, line management support, and intervention fit will be measured on managers, 10% of employees, and their union representatives (see Figure 2). At follow-up, around 4–6 months after baseline, the in-depth assessment will be repeated together with measures of the process and the actual actions at the IGLO levels that have been implemented (see Figure 2). Structural equation modeling will enable the test of CMO-configurations [52].

## 3. Discussion

Psychosocial risk management is crucial to ensure worker health and wellbeing and has received a huge attention at research and policy level in Europe as in most of member states [2,12]. Particularly in the “health and social work” sector, stress levels are higher compared to other sectors and employees are exposed to the highest levels of work intensity, demands, and adverse social behaviors [18].

The project testing the POI in psychosocial risk management is expected to improve our understanding of the intervention mechanisms and potential benefits of modifications in the work organization. Grounded on the INAIL’s methodology to managing psychosocial risks, we aim to explore potential benefits of modifications in the work organization that may lead to improvement in working conditions and job satisfaction [26]. In addition, this study includes a realist evaluation approach to answer the question of whether an intervention worked, as well as what worked for whom in which circumstances [48], through the examination of Context-Mechanisms-Outcome (CMO) configurations. We explore the potential efficacy of a POI on occupational health through five phases: 1. Preparation, 2. Preliminary Assessment, 3. In- depth assessment, 4. Actions and monitoring, and 5. Evaluation in two large Italian hospitals (around 7000 employees). We will collect pre-, during, and post-intervention surveys with the inclusion of psychosocial risks and job satisfaction at baseline and follow-up, according to literature and previous studies [53]. Data will be collected from managers, employees, and their representatives to provide a broad overview of specific risks factors in the work organization requiring intervention in light of having tailored or fitted the intervention to the organizational context. Moreover, this study benefits from the application of principles of a realist evaluation, since we will explore what works for whom in which circumstances. Such evaluation enables us to investigate how the process mechanisms, including managers’ commitment and involvement, employee participation, and tailoring for fit, influence the outcomes of the intervention, either facilitating or hindering its effectiveness [24,36]. In this study, we included job satisfaction as an outcome variable since this has shown a link with significant changes in working conditions during POIs [8,9] and a strong association with workers’ health and well-being [10]. While certain health and wellbeing outcomes are more appropriate for specific sectors and occupations (e.g., client-related burnout; emotional exhaustion), job satisfaction is relevant to all sectors and occupations. Taking into account the specificities of the study’s context, job satisfaction in healthcare workers is a parameter that has a great impact on quality of care, effectiveness, and commitment to work and, at the same time, on healthcare costs [54]. Some health outcomes (e.g., exhaustion, symptoms of distress) and further psychosocial risk aspects (e.g., rewards, job insecurity) should be also considered in the future to improve the study design.

This study has multiple strengths. It will be conducted in two large Italian hospitals, and this ensures having complex context with different areas and worksites and a potential applicability across a large number of healthcare settings. The division of employees in units (groups) in the POI allows a more realistic and effective assessment and action plan. The group division in large organizations helps in assessing the unique and more precise risk factors and identifying fitting actions, involving line managers of the single units, improving employees’ participation in the decision-making process. Manager involvement is important to link actions planned into the group to central actions and guarantee the effective changes in organizational procedures. Some actions needed at group level must indeed be driven by the organizational management, even if the role played by line managers in driving and pushing changes in behaviors and procedures at group level seems to be essential. In our view, groups also give us a better support in focusing on the IGLO model [46,47,55]. Starting from assessment findings for each group, the action plan will be developed by identifying actions needed at each level of the IGLO model (Individual, the Group, the Leader, and the Organizational level), incorporating both local and global perspectives.

The innovative focus of a participatory organizational-level intervention from the risk assessment to the managing of psychosocial risks in the healthcare sector including a realist evaluation, may allow, if successful, improvement of the existing strategies and support the improvement of the Italian policy framework. In Italy, the introduction of legislation on psychosocial risks assessment and management has had a good impact over time [6,7]. Nevertheless, more is needed to practically orient the management of psychosocial risks to reduce stress in terms of guidelines and tools to move from the assessment to the management of such risks.

## 4. Conclusions

This paper describes the background, study design, intervention approach, and evaluation methods for a POI in psychosocial risk management. This study also evaluates the feasibility and potential efficacy of an organizational approach to improve the working conditions and job satisfaction in the Healthcare. Findings will provide a structured process tailored to the Healthcare sector and practically support policy makers and institutional bodies in identifying specific needs of existing strategies and national policies on the management of psychosocial risks at work.

## Figures and Tables

**Table 1:** Indicators tailored to the healthcare sector to the INAIL Checklist.

Standard	Integrative *
Work-related Injuries	*Mortality Ratio*
Sick Leave Absences	*Complaints from patients and relatives*
Absences from Work	*Patient aggressions*
Left-over Vacation Days	*Internal transfers managed by the company*
Job Rotation	*Prevalence of employees from other or0ganization*
Turnover	*Prevalence of contingent employees*
Legal Actions/Disciplinary Sanctions	*Appropriate procedures for shift work*
Requests for Extraordinary	*Work availability at night*
Visits to the Occupational Physician	*Tiling and training procedures for newcomers*
Formal Records of Employees’ Complaints to the Company or to the Occupational Physician	*Analysis of training needs*
Work Environment and Work Equipment	*Organizational changes*
Task Planning	*Procedures for managing conflicts*
Work Load/Pattern of Work	*Procedures for managing conflicts with patients/relatives*
Working Hours	
Function and Organisational Culture	
Role Within the Organisation	
Career Path	
Decision-Making and Work Control	
Interpersonal Relationships at Work	
Home-Work Interface - Work/life Reconciliation	

* Indicators in italics are those developed and used on the Healthcare only. INAIL: Italian Workers Compensation Authority.

**Table 2:** Measures tailored to the healthcare sector to the Management Standards Indicator Tool.

Standard	Integrative **
Demands	*Work-family conflict*
Control	*Emotional burden*
Role	*Team/equip integration*
Support from colleagues	*Emotional dissonance*
Support from management	*Harassment and violence*
Relationships	*Ergonomic risk*
Change	*Organizational fairness*
	*Interaction with patients*

** Measures in italics are those developed and used on the Healthcare only.
